# Oxidative Damage of U937 Human Leukemic Cells Caused by Hydroxyl Radical Results in Singlet Oxygen Formation

**DOI:** 10.1371/journal.pone.0116958

**Published:** 2015-03-02

**Authors:** Marek Rác, Michal Křupka, Svatopluk Binder, Michaela Sedlářová, Zuzana Matušková, Milan Raška, Pavel Pospíšil

**Affiliations:** 1 Department of Biophysics, Centre of the Region Haná for Biotechnological and Agricultural Research, Faculty of Science, Palacký University, Šlechtitelů 11, 783 71, Olomouc, Czech Republic; 2 Department of Immunology, Faculty of Medicine and Dentistry, Palacký University, Hněvotínská 3, 775 15, Olomouc, Czech Republic; 3 Department of Medical Biophysics, Faculty of Medicine and Dentistry, Palacký University, Hněvotínská 3, 775 15, Olomouc, Czech Republic; 4 Department of Botany, Faculty of Science, Palacký University, Šlechtitelů 11, 783 71, Olomouc, Czech Republic; 5 Department of Pharmacology and Institute of Molecular and Translational Medicine, Faculty of Medicine and Dentistry, Palacký University, Hněvotínská 3, 775 15, Olomouc, Czech Republic; Martin-Luther-Universität Halle-Wittenberg, GERMANY

## Abstract

The exposure of human cells to oxidative stress leads to the oxidation of biomolecules such as lipids, proteins and nuclei acids. In this study, the oxidation of lipids, proteins and DNA was studied after the addition of hydrogen peroxide and Fenton reagent to cell suspension containing human leukemic monocyte lymphoma cell line U937. EPR spin-trapping data showed that the addition of hydrogen peroxide to the cell suspension formed hydroxyl radical via Fenton reaction mediated by endogenous metals. The malondialdehyde HPLC analysis showed no lipid peroxidation after the addition of hydrogen peroxide, whereas the Fenton reagent caused significant lipid peroxidation. The formation of protein carbonyls monitored by dot blot immunoassay and the DNA fragmentation measured by comet assay occurred after the addition of both hydrogen peroxide and Fenton reagent. Oxidative damage of biomolecules leads to the formation of singlet oxygen as conformed by EPR spin-trapping spectroscopy and the green fluorescence of singlet oxygen sensor green detected by confocal laser scanning microscopy. It is proposed here that singlet oxygen is formed by the decomposition of high-energy intermediates such as dioxetane or tetroxide formed by oxidative damage of biomolecules.

## Introduction

Reactive oxygen species (ROS) are continuously produced as byproducts of various metabolic pathways localized in the different cellular compartments. Superoxide anion radical (O_2_^•−^) is produced by electron leakage to molecular oxygen in mitochondria, endoplasmic reticulum, microbodies and cell walls [1,2]. The spontaneous and enzymatic dismutation of O_2_^•−^ results in the formation of hydrogen peroxide (H_2_O_2_), whereas the subsequent one-electron reduction of H_2_O_2_ by transition metal ions such as Fe^2+^, Cu^+^ and Zn^+^ results in the formation of hydroxyl radical (HO^•^) [3–7]. It is well known that ROS participate in various biochemical processes as signal transduction and defense against microbial pathogens [8,9]. Due to the highly positive redox potential of HO^•^/H_2_O redox couple (*E*_0_ *´*(HO^•^/H_2_O) = 2.3 V, pH 7), HO^•^ is highly reactive towards biomolecules such as lipids, proteins and nuclei acids. To eliminate the oxidative damage of biomolecules, the non-enzymatic and enzymatic antioxidant systems are engaged. When the concentration of ROS overwhelms the capacity of antioxidant system, HO^•^ causes the oxidative damage resulting in the structural and function modifications of lipids proteins and nuclei acids [10].

The formation of malondialdehyde (MDA), product of lipid peroxidation, is commonly used as a marker of oxidative damage of lipids [11]. It has been previously demonstrated that hydrogen abstraction from lipids mediated by HO^•^ results in the formation of lipid alkyl radical during the initiation of the lipid peroxidation (S1 Fig. panel A). In the next step, the unstable lipid alkyl radical reacts with molecular oxygen forming lipid peroxyl radical. During the propagation of lipid peroxidation, the lipid peroxyl radical reacts with free fatty acid forming another lipid alkyl radical and lipid hydroperoxide. In the termination step, lipid peroxyl radical forms cyclic peroxide and subsequently cyclic endoperoxide [10] know either to decompose to MDA and other products [12] or triplet excited carbonyl ^3^(R = O)* and lipid hydroxide. The triplet-singlet energy transfer from ^3^(R = O)* to molecular oxygen forms singlet oxygen (^1^O_2_). Alternatively, the recombination of two peroxyl radicals forms tetroxide which can decompose to ^1^O_2_ via Russell mechanism [13].

The protein carbonylation is commonly used as a marker of oxidative damage of proteins [14]. It is well established that the hydrogen abstraction from proteins by HO^•^ brings about the formation of protein alkyl radical known to interact with molecular oxygen forming protein peroxyl radical (S1 Fig. panel B). The second hydrogen abstraction by protein peroxyl radical from proteins leads to the formation of protein hydroperoxide known to be reduced to protein alkoxyl radical by transition metals such as Fe^2+^, Cu^+^ and Zn^+^. The β-scission of protein alkoxyl radical leads to the formation of protein carbonyls and protein alkyl radical [15]. It has been assumed that the recombination of protein peroxyl radicals forms tetroxide known to decompose to ^1^O_2_, protein carbonyls and protein hydroxide [16].

The DNA fragmentation is commonly used as a marker of oxidative damage of DNA [17]. It has been previously demonstrated that DNA oxidation is initiated by the hydrogen abstraction by HO^•^ from deoxyribose forming deoxyribose radical (S1 Fig. panel C). The subsequent radical reactions of deoxyribose radical lead to the rearrangement of the deoxyribose. The scission of the deoxyribose phosphate backbone results in the formation of DNA strand breaks [18]. The oxidative damage of DNA bases results in the alternation of the bases including thymine hydroperoxide. In the presence of metal ions or metalloproteins, thymine hydroperoxide can be reduced to thyimine peroxyl radical. The recombination of two thyimine peroxyl radicals through the Russell mechanism results in the formation of ^1^O_2_ [16].

In this study, the oxidative damage of biomolecules caused by the addition of H_2_O_2_ and Fenton reagent was examined. Whereas the lipid peroxidation was initiated solely after the addition of Fenton reagent, the protein carbonylation and DNA fragmentation occurred after the addition of both H_2_O_2_ and Fenton reagent. Evidence is provided that oxidative damage of lipids, proteins and DNA leads to the formation of ^1^O_2_ as confirmed by EPR spin-trapping spectroscopy and SOSG fluorescence using confocal laser scanning microscopy.

## Materials and Methods

### U937 cell culture

Human leukemic monocyte lymphoma cell line U937 was used. The culture is derived from histocytic lymphoma and is frequently used as a model of monocyte/macrophage cell lineage [19,20]. U937 cells were grown in RPMI-1640 supplemented with 2 mM L-glutamine, 10% FBS, antibiotics at 37°C in humidified 5% CO_2_ atmosphere. Viability of the cells was measured by Trypan Blue viability test. Subsequently, 10 μl of cell suspension was mixed with 10 μl of 0.4% Trypan Blue. Cells were counted by TC20 automated cell counter (Bio-Rad Laboratories, California, USA).

### EPR spin-trapping spectroscopy

EPR spin-trapping spectroscopy was used to monitor formation of HO^•^ and ^1^O_2_ in cell suspension. Hydroxyl radical was detected by a 4-pyridyl-1-oxide-*N*-*tert*-butylnitrone (POBN)/ethanol spin-trapping system [21]. Cell suspension was treated with 5 mM H_2_O_2_ or Fenton reagent (5 mM H_2_O_2_ and 1 mM FeSO_4_) in the presence of 50 mM POBN, 170 mM ethanol. Singlet oxygen was detected by hydrophilic spin trap compound TMPD (2, 2, 6, 6-Tetramethyl-4-piperidone) (Sigma) [22]. To eliminate impurity TMPD EPR signal TMPD was purified twice by vacuum distillation. Cell suspension was treated with 5 mM H_2_O_2_ or Fenton reagent (5 mM H_2_O_2_ and 1 mM FeSO_4_) in the presence of 100 mM TMPD and culture medium. Cell suspension previously exposed to H_2_O_2_ and Fenton reagent treatments were put in a glass capillary tube (Blaubrand intraMARK, Brand, Germany) and EPR spectra were recorded using an EPR spectrometer MiniScope MS400 (Magnettech GmbH, Berlin, Germany). The mixture of 10 mM molybdic acid and 10 mM H_2_O_2_ was used as a positive control for ^1^O_2_ generation. The TEMPONE EPR spectra were recorded in the presence of 50 mM TEMP immediately after the mixture was prepared. EPR conditions were as follows: microwave power, 10 mW; modulation amplitude, 1 G; modulation frequency, 100 kHz; sweep width, 100 G; scan rate, 1.62 G s-1, gain 500. To prevent overscaling of EPR signal, the gain was decreased to 100, when EPR signal was measured 30 min after the addition of Fenton reagent to the cell suspension. Simulation of EPR spectra was done using Winsim software freely available from the website of National Institute of Environmental Health Sciences.

### HPLC

The sample preparation and derivatization of malondialdehyde with 2,4-dinitrophenylhydrazine DNPH was performed as described previously [23] with some modifications. Cell suspension was treated with 5 mM H_2_O_2_ or Fenton reagent (5 mM H_2_O_2_ and 1 mM FeSO_4_) for 30 min were centrifuged for 20 min at 11000 x g and supernatant was removed. Pellet was stirred up in 200 μl of phosphate buffer saline (PBS). After this, cells were disrupted by sonication for 90 seconds. The sample with disrupted cells was centrifuged at 2000 x g for 10 min. The amount of 125 μl of supernatant was taken into the eppendorf vial and 25 μl of 6 M aqueous sodium hydroxide was added. This mixture was incubated in a 60°C water bath for 30 min to achieve alkaline hydrolysis of protein bound MDA. Then, protein was precipitated adding 62.5 μl of 35% (v/v) perchloric acid. The sample was vortexed and centrifuged at 16000 x g for 10 min. A volume of 125 μl of supernatant was put into the dark eppendorf vial and mixed with 1 μl DNPH prepared as a 50 mM solution in 50% sulphuric acid. This mixture was incubated in dark for 30 min at room temperature. An aliquot of 50 μl of this mixture was injected into the HPLC system. The samples were analyzed on the HPLC system (Shimadzu LC-20A Prominence, Kyoto, Japan) with UV detection at 310 nm. A Lichrospher 100 RP-18 column (4.0 x 250 mm) with 5 μm particle size (Merck, Germany) preceded by a Lichrospher precolumn of the same material as the stationary phase (4.0 x 4.0 mm) was used. Elution was performed isocraticaly with a mixture of 25 mM triethylamine adjusted to pH 3.5 and acetonitrile (50:50, v/v) at a flow rate of 1.5 ml/min at 35°C.

### Dot blot immunoassay

Proteins were isolated according to Pierce protocol with using RIPA buffer. Cell suspension treated with 5 mM H_2_O_2_ or Fenton reagent (5 mM H_2_O_2_ and 1 mM FeSO_4_) for 30 min were washed twice in cold PBS. Cold RIPA buffer was added and mixture was incubated on ice for 5 min. Lysate was centrifuged at 14 000 x g for 15 min at 4°C. Supernatant containing proteins was derivatized with 400 μM DNPH for 30 min. Samples were transferred onto the PVDF membrane, using Don-blot apparatus (BioRad). Membrane was blocked in SuperBlock blocking buffer (Thermo Scientific) for 1 h and afterwards was incubated with primary antibody (anti-dinitrophenyl-KLH, rabbit IgG fraction, biotin-labeled, Molecular Probes) overnight. Than membrane was washed PBS-T buffer (PBS + Tween 20) and incubated with streptavidin-HRP conjugate in blocking buffer for 1 h. After washing, the reaction was visualized by ECL chemistry using X-ray sensitive film.

### Comet assay

The DNA damage was studied by applying the comet assay. Microscope slides were firstly precoated with 1% HMP (high melting point) agarose (SERVA Electrophoresis), in distilled H_2_O and then placed in a drying oven at temperature of 60°C for at least 30 min. 85 μl of 1% HMP agarose in PBS was applied on the precoated slides covered with a cover slip. The slides were then placed in a refrigerator in order to enhance gelling of the agarose. Cell suspension treated with 5 mM H_2_O_2_ or Fenton reagent (5 mM H_2_O_2_ and 1 mM FeSO_4_) for 30 min was centrifuged (6 min, 1200 rpm) and cell pellet was dispersed in 20 μl of PBS and vortexed. 85 μl of 1% LMP (low melting point) agarose (Qbiogene) was added into this solution and 85 μl of this suspension was cast on the solidified agarose on the microscope slide (the cover slide was removed prior to cell inoculation on the gel) and covered by a new glass cover slip to form a thin layer and moved to the refrigerator again. After solidifying the cover slips were removed again and the microscope slides were immersed in a lysis buffer (2.5 M NaCl (Sigma-Aldrich), 100 mM EDTA (ethylenediaminetetraacetic acid) (Sigma-Aldrich), 10 mM Tris (tris(hydroxymethyl)aminomethane) (Sigma-Aldrich), 1% Triton X-100 (SERVA Electrophoresis), pH = 10) at 4°C for at least 1 h. After the lysis the slides were washed in distilled water to remove all salts and then placed in an electrophoretic tank and dipped in cool electrophoresis solution (300 mM NaOH (Sigma-Aldrich), 1 mM EDTA (Sigma-Aldrich) for 40 min. Electrophoresis was run at 0.8 V/cm and 350 mA for 20 min. After the electrophoresis the slides were rinsed 3x for 5min with neutralisation buffer (0.4 M Tris (Sigma-Aldrich), pH = 7.5) at 4°C. The samples were subsequently stained by SYBR Green (Invitrogen). Fifty randomly chosen cells from each sample was visualized using fluorescence microscope Olympus IX 70 with CCD camera. Computerized image analysis system (TriTek CometScore Freeware 1.5) was used to measure several comet parameters (tail length, tail moment, tail % DNA).

### Singlet oxygen detection by confocal laser scanning microscopy and image analysis

*In vivo* production of ^1^O_2_ was visualized by Singlet Oxygen Sensor Green (SOSG) reagent (Molecular Probes Inc., Eugene, OR, U.S.A.). 5mM SOSG stock solution was freshly prepared each time by adding 33 μL methanol to a 100 μg vial, and kept in darkness at +4°C until used during a half-day work session. Studied U937 cells were stained with the final concentration of 50 μM SOSG for 30 min, in darkness, at room temperature. At the beginning of incubation, cell suspension was supplemented with 5 mM H_2_O_2_ or Fenton reagent (5 mM H_2_O_2_ and 1 mM FeSO_4_). Negative controls were induced in the presence of 10 mM histidine. Following incubation, U937 cells were gently washed with 20 mM K-buffer, and consequently the SOSG fluorescence was measured by confocal laser scanning microscope, Fluorview 1000 confocal unit attached to IX 80 inverted microscope (Olympus Czech Group, Prague, Czech Republic). Microphotographs were taken in the transmitted light detection module (405 nm excitation and Nomarski DIC filters) combined with the fluorescence channel, representing the SOSG fluorescence (excitation by a 488 nm line of argon laser and detection by 505–525 nm emission filter set). At the start of each experiment, the proper intensity of lasers was checked according to an unstained sample. To evaluate differential ^1^O_2_ production during individual treatments, the intensities of SOSG fluorescence within confocal images were analyzed using Olympus FV10-ASW 3.0 Viewer software. The average intensity of SOSG fluorescence superior to the background and the percentage of U937 cells with signals were calculated from the SOSG fluorescence channel of five to ten representative microphotographs per variant.

### Statistical analysis

Statistical analysis was performed using software Statistika, version 12, (StatSoft CR s.r.o., Czech Republic). One-way ANOVA together with Post-hoc test at a significance level of 0.05 were used.

## Results

### Formation of hydroxyl radical detected by EPR spin-trapping spectroscopy

**Figure 1:**
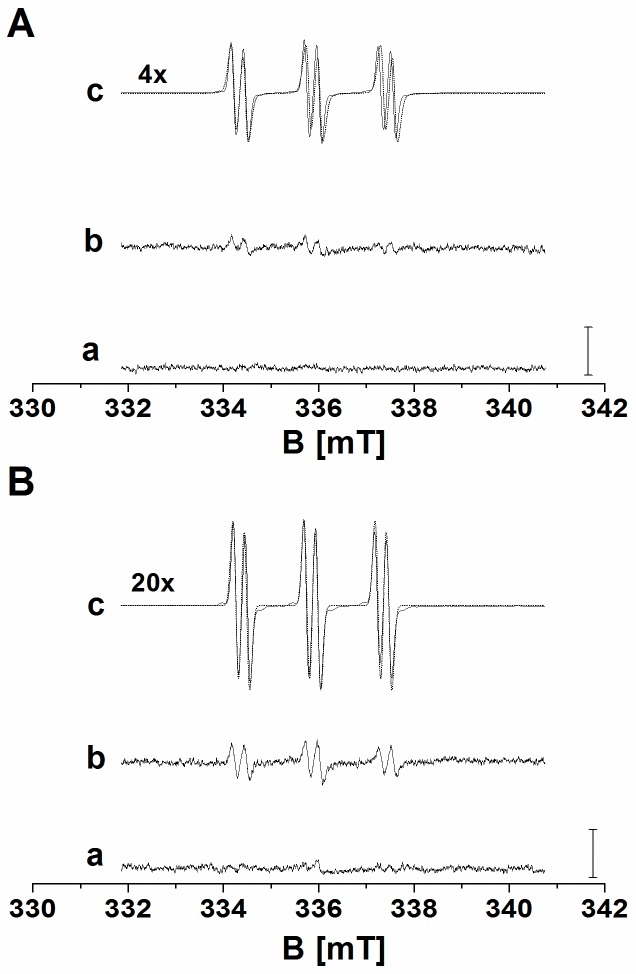
Detection of hydroxyl radical by EPR spin-trapping spectroscopy. EPR spectra of POBN-CH(CH_3_)OH adduct were detected 0 min (A) and 30 min (B) after the addition of H_2_O_2_ and Fenton reagent to the U937 cells. EPR POBN-CH(CH_3_)OH adduct spectra were measured in the control (trace a), the H_2_O_2_-treated (trace b) and the Fenton reagent-treated (trace c) U937 cells in the presence of 100 mM POBN/170 mM ethanol system. U937 cells were treated with 5 mM H_2_O_2_ (b) and Fenton reagent (5 mM H_2_O_2_ and 1 mM FeSO_4_) (c) for 0 min (A) and 30 min (B). In A and B, trace c (dotted line) shows the simulation of POBN-CH(CH_3_)OH EPR adduct signal using hyperfine coupling constants *a* ^N^ = 15,75 G, a^H^ = 2,40 G. Experimental EPR conditions were as follows: microwave power, 10 mW; modulation amplitude, 1 G; modulation frequency, 100 kHz; sweep width, 100 G; scan rate, 1.62 G s^−1^, gain 500. In B (trace c), to avoid overscaling of POBN-CH(CH_3_)OH adduct EPR signal, 50 mM POBN/170 mM ethanol system and gain 100 was used. Bars represent 2000 relative units.

To study the oxidative damage of biomolecules caused by ROS, H_2_O_2_ and Fenton reagent (H_2_O_2_ and FeSO_4_) were added to cell suspension. To confirm the formation of HO^•^ in cell suspension after the addition of H_2_O_2_ and Fenton reagent, EPR spin-trapping spectroscopy was used. The detection of HO^•^ was accomplished using POBN/ethanol spin-trapping system. It is well established that the interaction of HO^•^ with ethanol yields α-hydroxyethyl radical (CH(CH_3_)HO^•^) known to form a stable α-hydroxyethyl radical adduct of POBN (POBN-CH(CH_3_)OH adduct) by the interaction with POBN [19]. When POBN/ethanol spin-trapping system was added to the control cell suspension, no POBN-CH(CH_3_)OH adduct EPR spectrum was observed (Figs. 1A, trace a). The addition of H_2_O_2_ to the cell suspension forms small POBN-CH(CH_3_)OH adduct EPR signal (Fig. 1, trace b), while pronounced POBN-CH(CH_3_)OH adduct EPR signal was observed after the addition of Fenton reagent to the cell suspension (Fig. 1A, trace c). When POBN-CH(CH_3_)OH adduct EPR spectra were measured 30 min after the addition of H_2_O_2_ to the cell suspension (Fig. 1B, trace b), POBN-CH(CH_3_)OH adduct EPR signal was enhanced twice or three times as compared to POBN-CH(CH_3_)OH adduct EPR signal measured immediately after the addition of H_2_O_2_ (Fig. 1A, trace b). Similarly, POBN-CH(CH_3_)OH adduct EPR signal observed 30 min after the addition of Fenton reagent to the cell suspension increased ten times (Fig. 1B, trace c) as compared to POBN-CH(CH_3_)OH adduct EPR signal measured immediately after the addition of Fenton reagent (Fig. 1A, trace c). To confirm identification of POBN-CH(CH_3_)OH adduct EPR signal, simulation of experimental data was performed (Figs. 1A and B, trace c, dotted line). The best simulation of experimental data was accomplished using hyperfine coupling constants *a* ^N^ = 15.75 G, *a* ^H^ = 2.40 G known to be attributed to POBN-CH(CH_3_)OH adduct [21]. These observations reveal that HO^•^ is formed after the addition of H_2_O_2_ or Fenton reagent to the cell suspension with more pronounced effect observed after the addition of Fenton reagent.

### Cell viability

**Figure 2:**
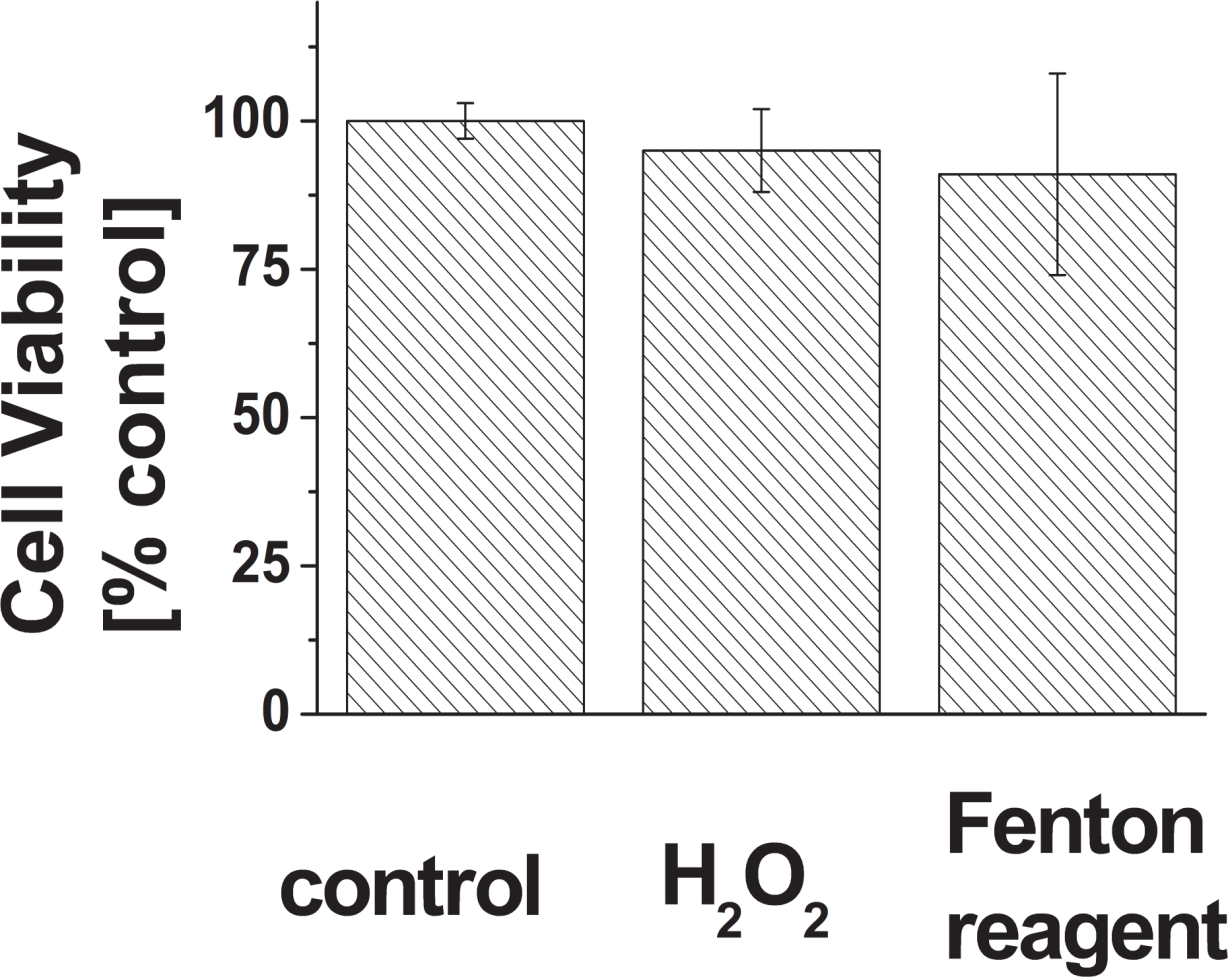
Determination of the U937 cell viability. The cell viability was determined 30 min after the addition of 5 mM H_2_O_2_ or Fenton reagent to the U937 cells. The results are normalized to control U937 cells. The data are presented as the mean and standard deviation of at least 3 measurements.

To test the effect of H_2_O_2_ and Fenton reagent addition to the U937 cells, the cell viability was counted using automated cell counter. Fig. 2 shows the viability of the cells 30 min after the addition of H_2_O_2_ or Fenton reagent compared to the control U937 cells. The results show that there is insignificant decrease in the cell viability in both samples indicating that almost all the cells are still alive after the treatment.

### Analysis of lipid peroxidation by MDA HPLC assay

**Figure 3:**
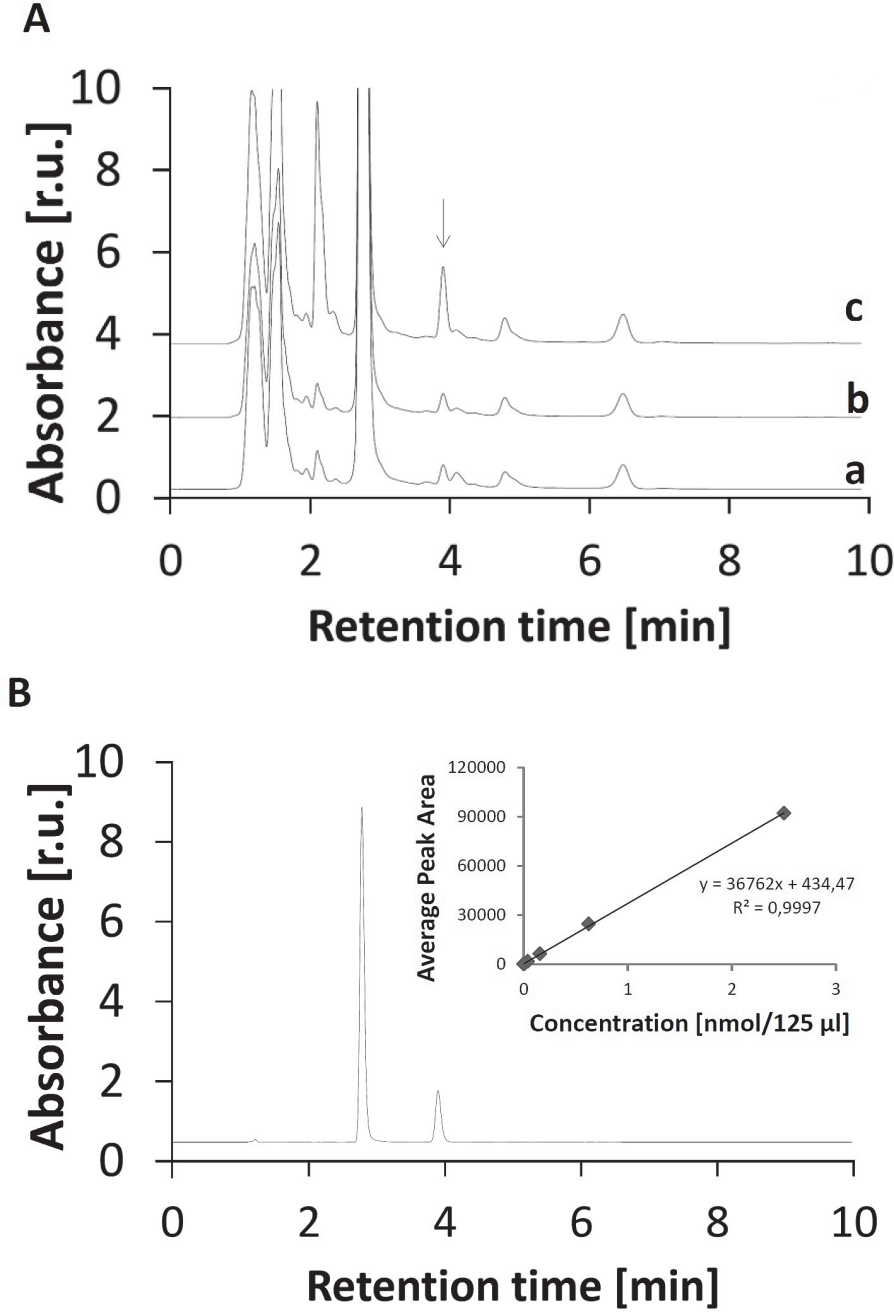
Detection of lipid peroxidation product malondialdehyde by HPLC analysis. The chromatogram of DNPH-MDA complex in U937 cells (A) and DNPH-MDA standard (B). In A, chromatogram of DNPH-MDA complex was measured in the control (trace a), the H_2_O_2_-treated (trace b) and the Fenton reagent-treated (trace c) U937 cells. The U937 cells were treated with 5 mM H_2_O_2_ (b) and Fenton reagent (5 mM H_2_O_2_ and 1 mM FeSO_4_) (c) for 30 min. After the treatment, lipids were separated from proteins and DNPH was added to lipids. In B, the chromatogram of DNPH-MDA standard shows the retention time of 3 min 50 s. The insert shows the dependence of average peak area on the concentration of DNPH-MDA standard. Based on the calibration curve, the concentrations of DNPH-MDA complex determined from calibration curve were as following: 0.029±0.003 nmol ml^−1^ (control), 0.030±0.003 (H_2_O_2_) and 0.09±0.02 nmol ml^−1^ (Fenton reagent). The coefficient of determination R^2^ was determined as 0.9997. Data are presented as mean values and standard deviations. The mean value represents the average value from at least three measurements.

Malondialdehyde, a product of lipid peroxidation, was detected to monitor oxidative damage of lipids caused by the addition of H_2_O_2_ and Fenton reagent to the cell suspension. An adaptation of a very rapid and simple isocratic reversed-phase HPLC separation of DNPH-MDA complex with absorption at 310 nm was used to examine the level of lipid peroxidation [23]. Fig. 3A shows the chromatogram of DNPH-MDA complex measured in the control (trace a), the H_2_O_2_-treated (trace b) and the Fenton reagent-treated (trace c) cell suspension. To estimate the retention time of DNPH-MDA complex, the chromatogram of DNPH-MDA standard was measured (Fig. 3B). The HPLC analysis of MDA-DNPH complex showed that the addition of H_2_O_2_ to the cell suspension caused no enhancement in peak area as compared to the control U937 cells. The peak area for the Fenton reagent-treated U937 cells was found three times higher compared to the peak area for the control and the H_2_O_2_-treated U937 cells. To quantify the concentration of MDA, standard calibration curve was obtained by plotting the peak area at 310 nm for various MDA concentrations (Fig. 3B, insert). The level of MDA in the control U937 cells and H_2_O_2_-treated U937 cells is 0.029±0.003 and 0.030±0.003 nmol ml^−1^, respectively, while in the Fenton reagent-treated U937 cells the level of MDA is 0.09±0.02 nmol ml^−1^. These observations reveal no difference in lipid peroxidation between the control and the H_2_O_2_-treated U937 cells while there is significant increase in lipid peroxidation in the Fenton reagent-treated U937 cells compared to the control U937 cells (p < 0.05).

### Analysis of protein *carbonylation* by dot blot immunoassay

**Figure 4:**
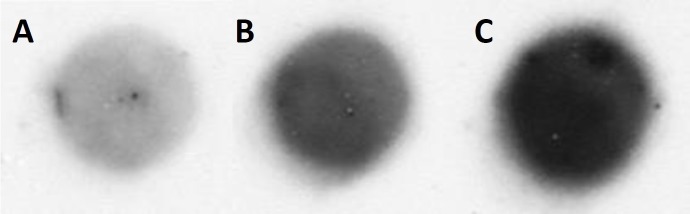
Detection of protein carbonyl compounds by dot blot immunoassay. Dot blot membrane in the control (A), the H_2_O_2_-treated (B) and the Fenton reagent-treated (C) U937 cells. The U937 cells were treated with 5 mM H_2_O_2_ (B) and Fenton reagent (5 mM H_2_O_2_ and 1 mM FeSO_4_) (C) for 30 min. After the treatment, the supernatant containing proteins were pipetted on the membrane and incubated with primary antibody (anti-dinitrophenyl-KLH, rabbit igG fraction, biotinilated) overnight. Furthermore the membrane was incubated with streptavidin-HRP conjugate for 1 h, developed with ECL and chemiluminescence signal was detected on X-ray film. The densitometry results are 0.5±0.2 (control), 1.4±0.2 (H_2_O_2_-treated), and 2.3±0.3 (Fenton reagent). Data are presented as mean values and standard deviations. The mean value represents the average value from at least three measurements.

To monitor the oxidative damage of proteins caused by the addition of H_2_O_2_ and Fenton reagent, the protein carbonyl levels were detected using 2,4-dinitrophenylhydrazine dot blot immunoassay. It is well established that carbonyl groups (aldehydes and ketones) are formed on the protein side chains during the oxidation of amino acid such as Pro, Arg, Lys and Thr. Due to their stability, protein carbonyl level is the most suitable marker of oxidative damage of proteins. The identification of protein carbonyl has been facilitated by the derivatization of the carbonyl group with DNPH forming DNP product and by the binding of specific anti-DNP antibodies allowing their detection by imunoblotting analysis. Fig. 4 demonstrates dot blot membrane obtained in the control (A), the H_2_O_2_-treated (B) and the Fenton reagent-treated (C) U937 cells. The addition of H_2_O_2_ to the U937 cells 30 min prior to the analysis attenuated protein carbonyl level (Fig. 4B) as compared to the control U937 cells (Fig. 4A) (p < 0.05). The most significant increase in protein carbonyl level was observed in the Fenton reagent-treated U937 cells (Fig. 4C) (p < 0.05). The quantification of dot blot membrane performed using densitometry shows that the enhancement in protein carbonyl level observed in the H_2_O_2_-treated and the Fenton reagent-treated U937 cells is twice and three times as compared to the control U937 cells, respectively. These observations indicate that the addition of H_2_O_2_ and Fenton reagent to the U937 cells resulted in the oxidative damage of proteins with more pronounced effect observed after the addition of Fenton reagent.

### Analysis of DNA fragmentation by comet assay

**Figure 5:**
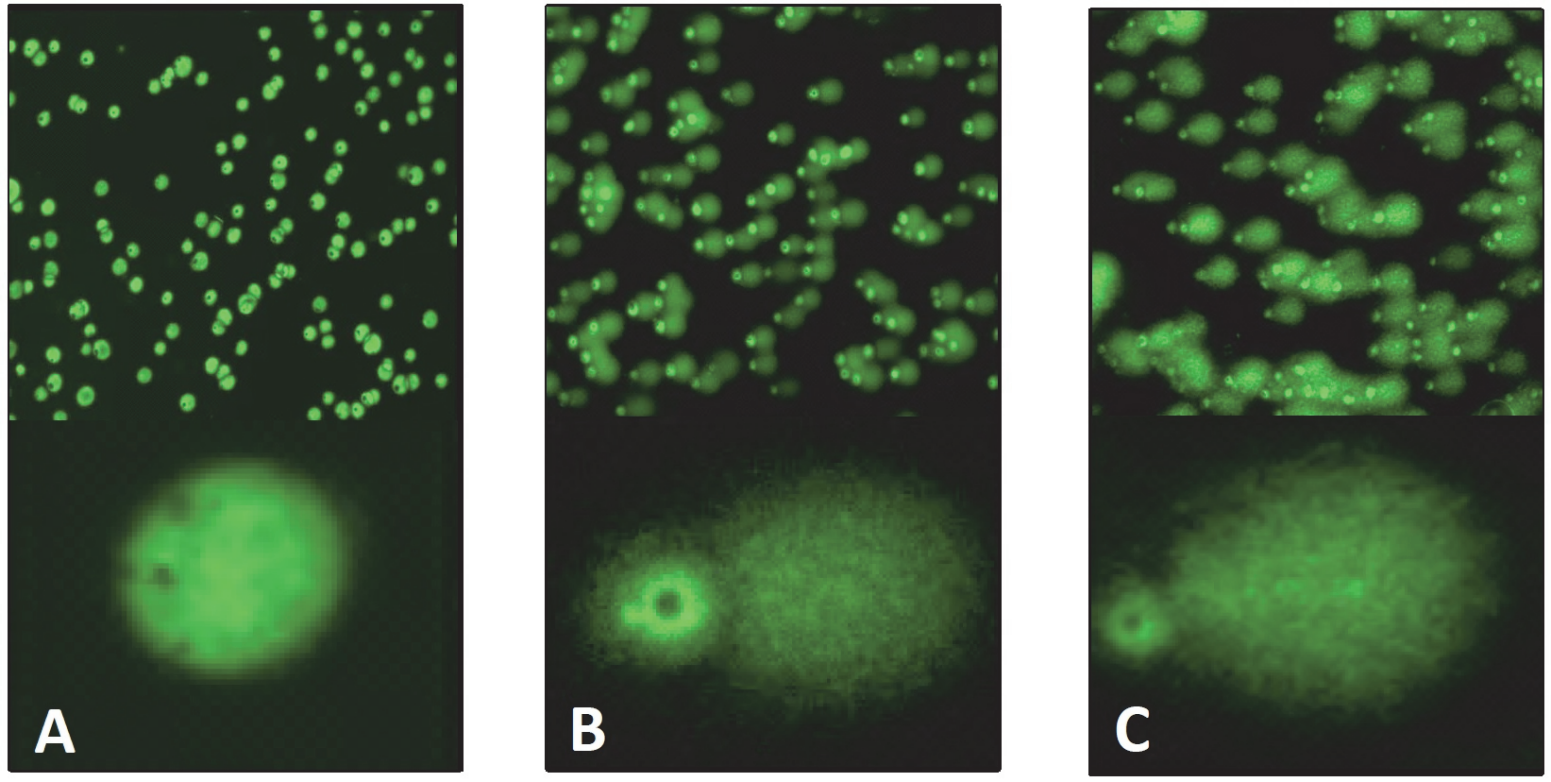
Analysis of DNA strand breaks by Comet assay. Comet assay of the control (A), the H_2_O_2_-treated (B) and the Fenton reagent-treated (C) U937 cells. The U937 cells were treated with 5 mM H_2_O_2_ (B) and Fenton reagent (5 mM H_2_O_2_ and 1 mM FeSO_4_) (C) for 30 min. After the treatment, U937 cells were stained by SYBR Green.

In order to examine the oxidative damage of DNA caused by the addition of H_2_O_2_ and Fenton reagent to the cell suspension, the DNA fragmentation was measured by comet assay (single cell electrophoresis). In this method, the comet tail which represents the fragmented DNA from U937 cells migrates towards the anode. The visualization of DNA from tail and head stained by SYBR Green was performed by fluorescence microscopy. Fig. 5 shows the comet assay images of the control (A), the H_2_O_2_-treated (B) and the Fenton reagent-treated (C) U937 cells. In the control U937 cells, intact comet heads were observed, whereas heads and tails were distinguished in the H_2_O_2_-treated and the Fenton reagent-treated U937 cells. Table 1 summarizes the parameters of comet, head and tail determined by the image analysis in the control, the H_2_O_2_-treated and the Fenton reagent-treated U937 cells. Statistical analysis reveals that most of the parameters are significantly different in the H_2_O_2_-treated and the Fenton reagent-treated U937 cells compared to the control U937 cells (p < 0.05), whereas no significant differences were observed between the H_2_O_2_-treated and the Fenton reagent-treated U937 cells. These observations indicate that the addition of both H_2_O_2_ and Fenton reagent to U937 caused the fragmentation of DNA with non-significantly higher effect observed after the addition of Fenton reagent.

**Table 1:** Analysis of the medians of comet, head, and tail parameters in the control, the H_2_O_2_-treated and the Fenton reagent-treated U937 cells.

Comet					
	Length [μm]	Height [μm]	Area [μm^2^]	Intensity [r.u.]	Mean Intensity [r.u.]
**Control**	20 ± 2	18 ± 2	301 ± 71	71506 ± 18571	51 ± 5
**H_2_O_2_**	44 ± 2	24 ± 2	762 ± 66	99182 ± 7767	27 ± 1
**Fenton**	51 ± 6	25 ± 3	301 ± 71	123451 ± 18571	51 ± 5
Head					
	Diameter [μm]	%DNA	Area [μm^2^]	Intensity [r.u.]	Mean Intensity [r.u.]
**Control**	18 ± 2	91 ± 4	262 ± 48	65320 ± 16978	52 ± 7
**H_2_O_2_**	13 ± 1	30 ± 2	178 ± 15	27219 ± 1385	31 ± 5
**Fenton**	10 ± 1	21 ± 10	141 ± 32	24705 ± 7742	31 ± 2
Tail					
	Length [μm]	%DNA	Area [μm^2^]	Intensity [r.u.]	Mean Intensity [r.u.]
**Control**	2 ± 1	8 ± 4	31 ± 21	5888 ± 3993	42 ± 9
**H_2_O_2_**	30 ± 3	69 ± 3	524 ± 36	64238 ± 9192	26 ± 1
**Fenton**	39 ± 8	78 ± 10	659 ± 161	94581 ± 32988	29 ± 2

Data are presented as mean values and standard deviations. The mean value represents the average value from at least three measurements.

### Formation of singlet oxygen detected by EPR spin-trapping spectroscopy

**Figure 6:**
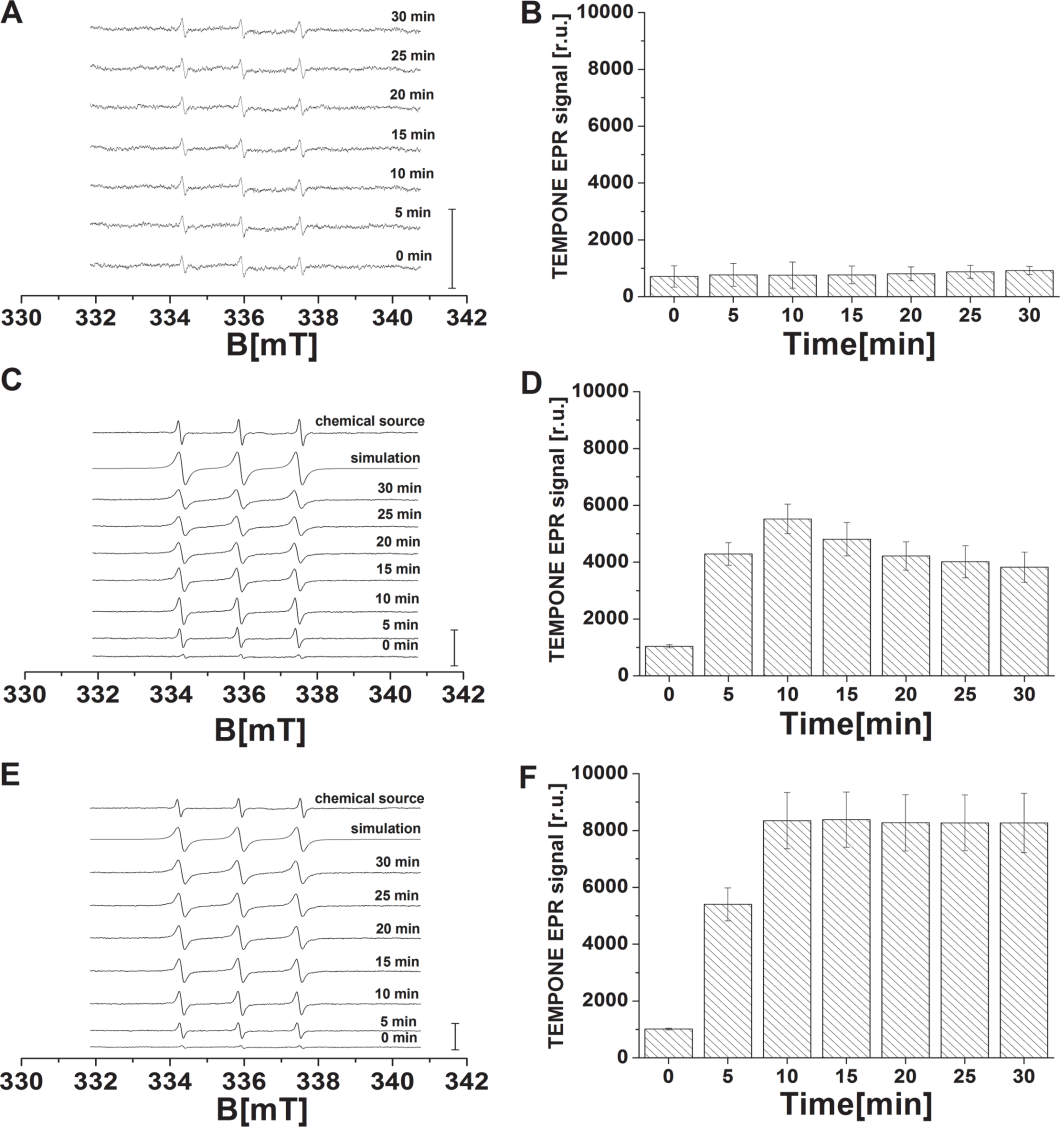
Detection of singlet oxygen by EPR spin-trapping spectroscopy. TEMPONE EPR spectra were measured in the control (A and B), the H_2_O_2_-treated (C and D) and the Fenton reagent-treated (E and F) U937 cells in the presence of 100 mM TEMPD. U937 cells were treated with no addition (A), 5 mM H_2_O_2_ (C) and Fenton reagent (5 mM H_2_O_2_ and 1 mM FeSO_4_) (E) for a period indicated in Fig. In C and E, top traces show the simulation of TEMPONE EPR signal using hyperfine coupling constants *a* ^N^ = 16 G. Chemical source of ^1^O_2_ (10 mM molybdic acid + 10 mM H_2_O_2_ measured right after the preparation) was used as a positive control (C,E). Bar graphs represent the hight of the middle peak of TEMPONE EPR signal in the control (B), the H_2_O_2_-treated (D) and the Fenton reagent-treated (F) U937 cells. Experimental EPR conditions were as follows: microwave power, 10 mW; modulation amplitude, 1 G; modulation frequency, 100 kHz; sweep width, 100 G; scan rate, 1.62 G s-1, gain 500. Bars represent 4000 (A) and 8000 (C and E) relative units. Data are presented as mean values and standard deviations. The mean value represent the average value from at least three measurements.

To study whether oxidative damage of biomolecules generates ^1^O_2_, EPR spin-trapping technique was used to monitor the formation of ^1^O_2_ in the H_2_O_2_-treated and the Fenton reagent-treated cell suspension. The spin-trapping was accomplished by utilizing the oxidation of hydrophilic diamagnetic TMPD by ^1^O_2_ know to yield paramagnetic 2, 2, 6, 6-tetramethyl-4-piperidone-1-oxyl (TEMPONE). The addition of TMPD to the control cell suspension results in the appearance of negligible TEMPONE EPR signal (Fig. 6A). Fig. 6B shows that negligible TEMPONE EPR signal was stable over the whole measured period. When TEMPONE EPR spectra were measured after the addition of H_2_O_2_ to the cell suspension, TEMPONE EPR signal was observed (Fig. 6C). Fig. 6D shows that TEMPONE EPR signal increases within 10 min followed by gradual decrease. The gradual decrease of TEMPONE EPR signal is likely caused by the oxidation of TEMPONE resulting in the formation of EPR silent oxidized TEMPONE. These observations indicate that ^1^O_2_ is formed after the addition of H_2_O_2_ and Fenton reagent to the cell suspension. When TEMPONE EPR spectra were measured after the addition Fenton reagent, significant TEMPONE EPR signal was observed (Fig. 6E). Fig. 6F shows that TEMPONE EPR signal increases within 10 min followed by steady-state level. Comparison of TEMPONE EPR signals showed TEMPONE EPR signals measured after the addition of Fenton reagent is higher compared to TEMPONE EPR signals measured after the addition of H_2_O_2_. Statistical analysis of the data revealed there is significant difference between control and treated samples after 5 min of treatment (p < 0.05). The results from control, H_2_O_2_ treated and Fenton treated samples obtained 10–30 min of treatment were found to be significantly different (p < 0.05). Fig. 6 (C and E) show the simulation of TEMPONE EPR signal using hyperfine coupling constants *a* ^N^ = 16 G, known to be attributed to TEMPONE [24]. These findings reveal the formation of ^1^O_2_ after the addition of H_2_O_2_ or Fenton reagent to the cell suspension with more pronounced effect observed after the addition of Fenton reagent.

### Formation of singlet oxygen detected by confocal laser scanning microscopy

**Figure 7:**
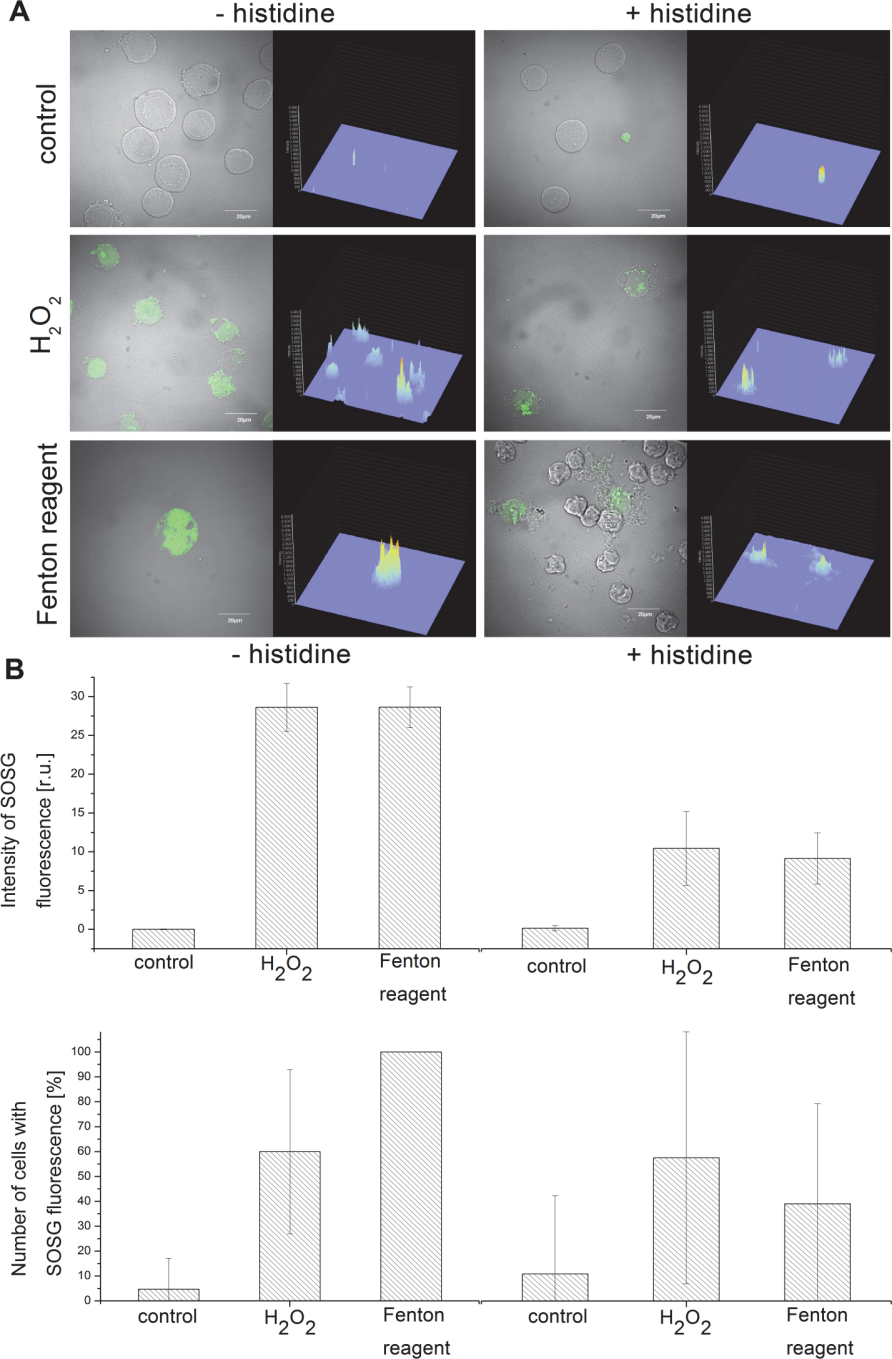
Singlet oxygen imaging by confocal laser scanning microscopy. The SOSG fluorescence from U937 cells was measured after the addition of 5 mM H_2_O_2_ and Fenton reagent (5 mM H_2_O_2_ and 1 mM FeSO_4_) by confocal laser scanning microscopy. 50 μM SOSG was added 30 min prior to the data collection to the U937 cells. In A, left column shows the combination of Nomarski DIC and SOSG fluoresencence (λ_em_ = 505–525 nm) channels and right column the integral distribution of SOSG fluorescence. In B, the intensity of SOSG fluorescence (upper graph) and the percentage of cells showing the SOSG fluorescence (lower graph) are shown. Data are presented as mean values and standard deviations. The mean value represents the average value from five to ten representative microphotographs per variant.

To visualize the formation of ^1^O_2_ in U937 cells after the addition of H_2_O_2_ and Fenton reagent to the cell suspension, the SOSG fluorescence was measured by the confocal laser scanning microscopy (Fig. 7A). In order to quantify the ^1^O_2_ formation, the intensity of SOSG fluorescence and the percentage of cells showing the SOSG fluorescence were evaluated (Fig. 7B). Negligible SOSG fluorescence was recorded in 5% of the control U937 cells. In order to confirm that the SOSG fluorescence origins from the interaction of SOSG with ^1^O_2_, the effect of histidine on the SOSG fluorescence was measured. No difference in SOSG fluorescence was found in the control U937 cells incubated in the presence of histidine while the number of cells with SOSG fluorescence was 10%. No changes in cell morphology were found in the control U937 cells. The addition of H_2_O_2_ to the U937 cells resulted in a pronounced SOSG fluorescence observed in 60% of cells. SOSG fluorescence was localized in cytoplasm, nucleus and probably mitochondria. U937 cells were changed in shape, increased volume and became strongly vacuolized. The effect of H_2_O_2_ was significantly decreased in the presence of histidine (3 times lower signal in 53% of cells) (p < 0.05). The addition of Fenton reagent to the U937 cells caused SOSG fluorescence in all cells, though the signal intensity was comparable to that in the H_2_O_2_-treated cells. The volume and shape of cells were dramatically changed, some of them ruptured. Percentage of cells producing SOSG fluorescence was diminished by a histidine addition to 36%, whereas the intensity of SOSG fluorescence was slightly lower than in the H_2_O_2_-treated U937 cells in the presence of histidine.

## Discussion

In this study, the oxidation of biomolecules such as lipids, proteins and nuclei acids by HO^•^ was studied after the addition of H_2_O_2_ and Fenton reagent to the cell suspension. The experimental data provided showed that hydrogen abstraction by HO^•^ initiates a cascade of oxidative reactions leading to lipid peroxidation, protein carbonylation and DNA fragmentation (Figs. 3–5). In these processes, hydrogen abstraction by HO^•^ from biomolecules forms alkyl radical known to from peroxyl radical by the interaction with molecular oxygen. Peroxyl radical abstracts hydrogen from another biomolecule resulting in the formation of hydroperoxide and alkyl radical [10]. Experimental evidence obtained by EPR spin-trapping spectroscopy (Fig. 6) and SOSG fluorescence using confocal laser scanning microscopy (Fig. 7) showed that the oxidative damage of lipids, proteins and nucleic acids leads to the formation of ^1^O_2_. The decomposition of high-energy intermediates such as dioxetane or tetroxide is proposed as a plausible mechanism for ^1^O_2_ formation caused by the oxidative damage of biomolecules.

### Hydroxyl radical formation

EPR spin-trapping data showed that the addition of H_2_O_2_ (Figs. 1A and B, trace b) and Fenton reagent (Figs. 1A and B, trace c) to the cell suspension caused formation of HO^•^ via Fenton reaction. In this reaction, a reduced metal ion reacts with H_2_O_2_ forming an oxidized metal ion and HO^•^ [25]. Whereas after the addition of H_2_O_2_ to the cell suspension HO^•^ is formed by the reaction of H_2_O_2_ with endogenous metal ions, in Fenton reagent, HO^•^ is formed by the reaction of H_2_O_2_ with exogenous iron ions. Several types of endogenous metal ions such as iron, copper, manganese, zinc, chromium, cobalt, nickel and vanadium have been shown to reduce H_2_O_2_ to HO^•^. It is well known that endogenous iron ions are coordinated to active enzyme site in metalloproteins or stored in a ubiquitous protein called ferritin [25]. Due to the lower concentration of free endogenous metal ions compared to the concentration of iron in the Fenton reagent, the formation of HO^•^ after the addition of H_2_O_2_ is significantly lower than after the addition of Fenton reagent (Fig. 1). Due to the highly positive redox potential of HO^•^/H_2_O redox couple (*E*_0_ *´*(HO^•^/H_2_O) = 2.3 V, pH 7), HO^•^ is highly reactive towards lipids, proteins and nuclei acids. As the high reactivity of HO^•^ restricts diffusion of HO^•^ from the site of its formation, HO^•^ formed by the reaction of H_2_O_2_ with endogenous metal ions oxidizes biomolecules solely in the close proximity to the site of its formation [25]. Contrary, the oxidation of biomolecules by HO^•^ formed by the reaction of H_2_O_2_ with exogenous iron ions is not site-specific and therefore HO^•^ can react with a broader range of biomolecules in the cell.

### Oxidative damage initiated by hydroxyl radical

**Lipid peroxidation**. The HPLC analysis of MDA-DNPH complex showed that the addition of H_2_O_2_ to the cell suspension has no effect on lipids, whereas the addition of Fenton reagent to the cell suspension caused pronounced lipid peroxidation in the U937 cells (Fig. 3). The observation that no lipid peroxidation was observed after the addition of H_2_O_2_ to the cell suspension reveals that H_2_O_2_ has no capability to directly initiate lipid peroxidation [26]. Due to the fact that no metal ions are bound to lipids, the formation of HO^•^ by reduction of H_2_O_2_ by endogenous metals via Fenton reaction is unlikely [10]. The observation that lipid peroxidation was detected after the addition of Fenton reagent to the cell suspension indicates that HO^•^ initiates lipid peroxidation by the abstraction of hydrogen from lipid and the formation of lipid alkyl radical. In the propagation step, lipid alkyl radical reacts with molecular oxygen forming peroxyl radical known to react with another lipid resulting in the formation of another lipid alkyl radical. In the termination step, lipid peroxidation is terminated either by the cyclization of peroxyl radical to cyclic endoperoxide decomposing to MDA or by the recombination of two peroxyl radicals producing tetroxide decomposing to ^1^O_2_ via Russell mechanism [13].

**Protein** carbonylation. The addition of H_2_O_2_ and Fenton reagent to the cell suspension resulted in the oxidative damage of proteins in the U937 cells with significantly higher effect observed after the addition of Fenton reagent (Fig. 4). These results correlate with the EPR spin-trapping data which showed significantly higher formation of HO^•^ in the cell suspension treated with Fenton reagent. It is well known that protein carbonylation occurs both on the protein side chain and the protein backbone. On the protein side chain, the carbonylation of protein proceeds via direct oxidation or chain reaction. The direct oxidation of protein side chains (especially of Pro, Arg, Lys, and Thr) results in the formation of carbonyl groups [27]. In the chain reaction, the hydrogen abstraction from an amino acid residue by HO^•^ results in the formation of protein alkyl radical. Protein alkyl radical can subsequently react with molecular oxygen forming protein peroxyl radical. The recombination of two peroxyl radicals gives a raise to unstable tetroxide, which decomposes to two alkoxyl radicals. Once the alkoxyl radical is formed, it can undergo through rearrangement of electron resulting in the formation of carbonyl radical and carbonyl compound on the protein side chain. On the protein backbone, the formation of alkoxyl radical can also result in the oxidative cleavage of protein backbone by either the α-amidation pathway or by oxidation of glutamyl side chains leading to the formation of carbonyl groups [15].

**DNA fragmentation**. The addition of both H_2_O_2_ and Fenton reagent to the cell suspension caused the fragmentation of DNA in U937 cells with non-significantly higher effect observed after the addition of Fenton reagent (Fig. 5). It was previously reported that no DNA strand breaks were formed during the interaction of H_2_O_2_ and isolated DNA [28], whereas HO^•^ formed via Fenton reaction forms DNA strand breaks [29,30]. At least five main classes of oxidative damage caused by HO^•^ have been proposed including oxidized bases, abasic sites, DNA-DNA intra-strand adducts, DNA strand breaks and DNA-protein cross-links. It has been proposed that the extent of DNA strand breaking by HO^•^ is governed by the accessible surface areas of the hydrogen atoms of the DNA backbone [31].

### Singlet oxygen formation caused by oxidative damage of U937 cells

In this study, EPR spin-trapping spectroscopy was used to monitor the formation of ^1^O_2_ in U937 cells caused by HO^•^. When spin-trapping was performed by the oxidation of lipophilic 2,2,6,6-tetramethylpiperidine/ethanol (TEMP/ethanol) system by ^1^O_2_ resulting in the formation of 2,2,6,6-tetramethylpiperidine-1-oxyl (TEMPO), significant TEMPO EPR signal was detected (data not shown). Based on the observation that H_2_O_2_ was found to oxidize TEMP/ethanol system, the use of lipophilic TEMP/ethanol system was feasible. To prevent the oxidation of TEMP/ethanol system by H_2_O_2_, the spin-trapping was accomplished by utilizing the oxidation of hydrophilic TMPD by ^1^O_2_ which yields 2, 2, 6, 6-tetramethyl-4-piperidone-1-oxyl (TEMPONE). The analysis of TEMPONE EPR spin-trapping data showed that ^1^O_2_ was detected after the addition of H_2_O_2_ and Fenton reagent to U9370 cells (Fig. 6). The kinetics of ^1^O_2_ formation revealed that the formation of ^1^O_2_ is predominantly during the first 10 min in both the H_2_O_2_-treated (Fig. 6D) and the Fenton reagent-treated (Fig. 6F) cell suspension. The slow decay in the H_2_O_2_-treated cell suspension was most probably caused by the oxidation of TEMPONE resulting in the formation of EPR silent TEMPONE. The instability of TEMPONE limits the accurate quantification of ^1^O_2_ formation over the longer time period. The overall formation of ^1^O_2_ in the Fenton reagent-treated sample was double compared to the H_2_O_2_-treated sample. In order to visualize the formation of ^1^O_2_ in the U937 cells, the SOSG fluorescence was measured (Fig. 6). It has been previously demonstrated that SOSG is unable to penetrate into the CNE2 cells [32]. However it has been demonstrated that SOSG can penetrate HeLA cells if cultivated in a special medium lacking proteins [33]. Our study showed that SOSG can penetrate through the plasma membrane of U937 cells with no such kind of limitations. The formation of ^1^O_2_ within the U937 cells was about the same in the H_2_O_2_-treated and the Fenton reagent-treated cell suspension, while the amount of cells in which ^1^O_2_ was produced was double in the Fenton reagent-treated cell suspension compared to the H_2_O_2_-treated cell suspension.

The formation of ^1^O_2_ detected both by EPR spin-trapping spectroscopy and SOSG fluorescence using confocal laser scanning microscopy occurs in the parallel to lipid peroxidation, protein carbonylation and DNA fragmentation. These observations indicate that the formation of ^1^O_2_ is closely associated with damage of lipids, proteins and DNA. Several lines of evidence have been provided that ^1^O_2_ is formed by decomposition of high-energy intermediates such as dioxetane and tetroxide formed during the oxidative damage of lipids, proteins and DNA [16,34].

**Singlet oxygen by dioxetane decomposition**. Dioxetane is formed via two different mechanisms comprising the cycloaddition of ^1^O_2_ to biomolecules or the cyclization of peroxyl radical [35]. Here, it is proposed that the formation of dioxetane takes place during the physiological and pathological conditions since the concentration of peroxyl radical is unlikely high enough for the formation of tetroxide via recombination of peroxyl radicals. Unstable dioxetane subsequently undergoes the decomposition to ^3^(R = O)* and organic hydroxide. The excitation energy from ^3^(R = O)* is transferred to molecular oxygen resulting in the formation of ^1^O_2_ [36]. It is suggested here that the decomposition of dioxetane is responsible for the ^1^O_2_ formation shortly after the addition of H_2_O_2_ and Fenton reagent to the cell suspension.

**Singlet oxygen by tetroxide decomposition**. It was previously reported that lipid and DNA hydroperoxides can be decomposed to peroxyl radical in the presence of metal ions, cytochrome c, peroxynitrite, chloroperoxide, and hypochlorous acid [37,38]. It has been previously suggested that the recombination of two peroxyl radicals occurs mainly in chemical system or under high oxidative damage conditions [16]. The recombination of two peroxyl radicals forms unstable tetroxide which decompose either to ^1^O_2_, carbonyl and organic hydroxide or molecular oxygen, ^3^(R = O)* and organic hydroxide. Furthermore the ^3^(R = O)* can react with the molecular oxygen forming ^1^O_2_ [16]. It has been established in the chemical system that the formation of ^1^O_2_ by the recombination of peroxyl radicals is 3–4 magnitude higher compared to the formation of ^3^(C = O)* formed the recombination of peroxyl radicals [39]. It is suggested here that the decomposition of hydroperoxides takes place later after the addition of H_2_O_2_ and Fenton reagent to the cell suspension due to the long lifetime of organic hydroperoxides.

## Supporting Information

S1 Fig The formation of markers of oxidative damage to biomolecules. Panel A: Formation of MDA during lipid peroxidation. In the first step, HO^•^ abstracts the hydrogen atom from lipid resulting in the formation of alkyl radical (reaction 1). Subsequent reaction of alkyl radical with molecular oxygen give raise to the peroxyl radical (reaction 2). Peroxyl radical undergoes cyclization to form cyclic peroxide (reaction 3) and consequently cyclic endoperoxide (reaction 4) known to decompose to alkyl radical and MDA (reaction 5). Panel B: Formation of protein carbonyl by β-scission of protein alkoxyl radical. The abstraction of hydrogen from carbonyl by HO^•^ results in the formation of protein alkyl radical (reaction 1) known to interact with molecular oxygen forming protein peroxyl radical (reaction 2). The second hydrogen abstraction by protein peroxyl radical from proteins leads to the formation of protein hydroperoxide (reaction 3) known to be reduced to protein alkoxyl radical by Fe^2+^ (reaction 4). The β-scission of protein alkoxyl radical leads to the formation of protein carbonyls and protein alkyl radical (reaction 5). Panel 3: DNA strand break initiated by HO^•^. Hydrogen abstraction from deoxyribose forms deoxyribose radical (reaction 1) resulting in the instability of the deoxyribose phosphate backbone leading to the strand break (reaction 2).
(TIF)
