# Supplementary figures and images for: Oxidative Damage of U937 Human Leukemic Cells Caused by Hydroxyl Radical Results in Singlet Oxygen Formation
Source: PLoS One. 2015 Mar 2;10(3):e0116958. doi: 10.1371/journal.pone.0116958 (PMC4346403; doi:10.1371/journal.pone.0116958)

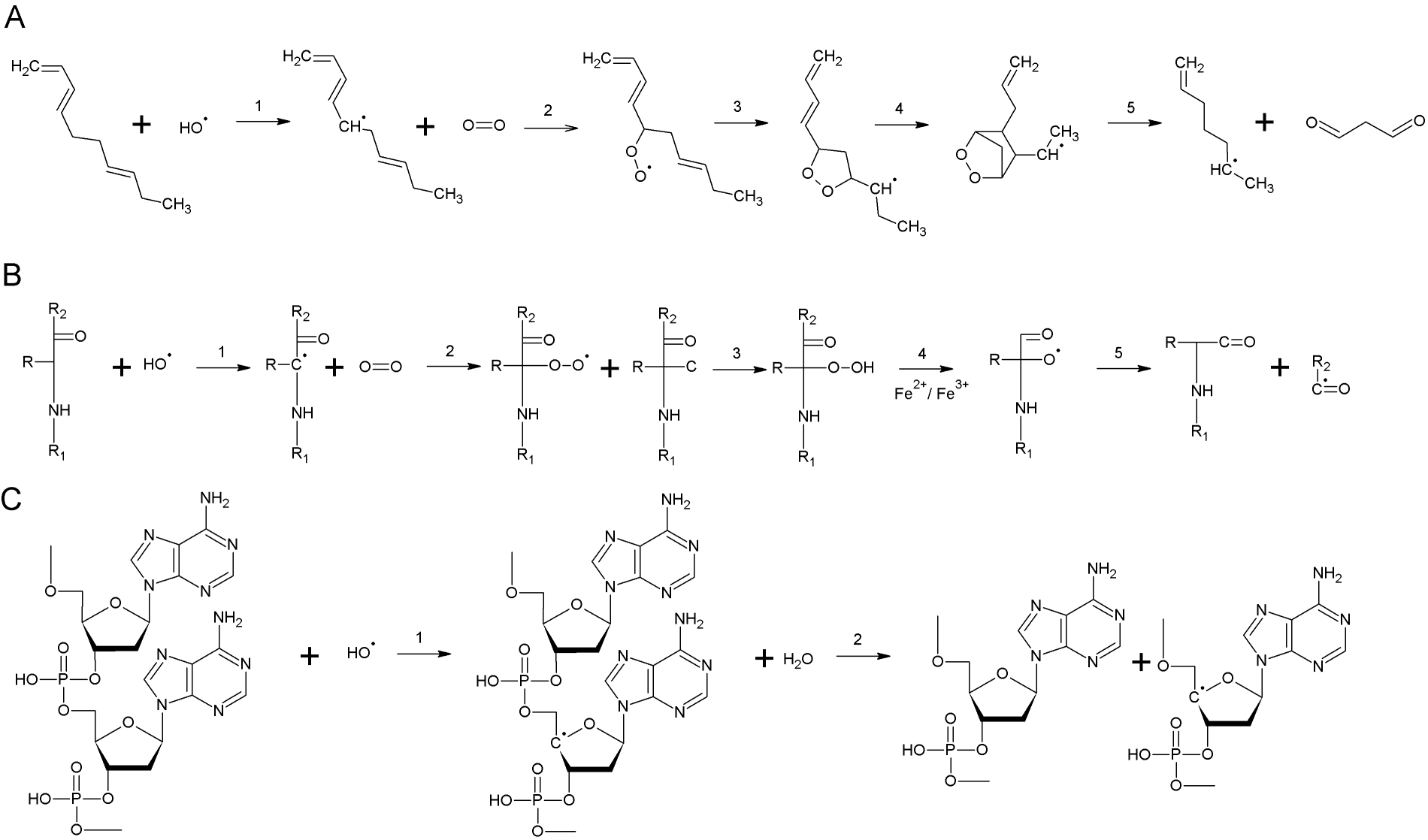

Supplement: S1 Fig — Panel A: Formation of MDA during lipid peroxidation. In the first step, HO• abstracts the hydrogen atom from lipid resulting in the formation of alkyl radical (reaction 1). Subsequent reaction of alkyl radical with molecular oxygen give raise to the peroxyl radical (reaction 2). Peroxyl radical undergoes cyclization to form cyclic peroxide (reaction 3) and consequently cyclic endoperoxide (reaction 4) known to decompose to alkyl radical and MDA (reaction 5). Panel B: Formation of protein carbonyl by β-scission of protein alkoxyl radical. The abstraction of hydrogen from carbonyl by HO• results in the formation of protein alkyl radical (reaction 1) known to interact with molecular oxygen forming protein peroxyl radical (reaction 2). The second hydrogen abstraction by protein peroxyl radical from proteins leads to the formation of protein hydroperoxide (reaction 3) known to be reduced to protein alkoxyl radical by Fe2+ (reaction 4). The β-scission of protein alkoxyl radical leads to the formation of protein carbonyls and protein alkyl radical (reaction 5). Panel 3: DNA strand break initiated by HO•. Hydrogen abstraction from deoxyribose forms deoxyribose radical (reaction 1) resulting in the instability of the deoxyribose phosphate backbone leading to the strand break (reaction 2). (TIF) [file pone.0116958.s001.tif]
